# Recent Developments in Hyperspectral Imaging for Assessment of Food Quality and Safety

**DOI:** 10.3390/s140407248

**Published:** 2014-04-22

**Authors:** Hui Huang, Li Liu, Michael O. Ngadi

**Affiliations:** Department of Bioresource Engineering, McGill University, Macdonald Campus, 21,111 Lakeshore Road, Ste-Anne-de-Bellevue, QC H9X 3V9, Canada; E-Mails: hui.huang3@mail.mcgill.ca (H.H.); li.liu5@mcgill.ca (L.L.)

**Keywords:** hyperspectral imaging, image processing, food quality and safety

## Abstract

Hyperspectral imaging which combines imaging and spectroscopic technology is rapidly gaining ground as a non-destructive, real-time detection tool for food quality and safety assessment. Hyperspectral imaging could be used to simultaneously obtain large amounts of spatial and spectral information on the objects being studied. This paper provides a comprehensive review on the recent development of hyperspectral imaging applications in food and food products. The potential and future work of hyperspectral imaging for food quality and safety control is also discussed.

## 1. Introduction

With the current growing need for low production costs and high efficiency, the food industry is faced with a number of challenges, including maintenance of high-quality standards and assurance of food safety while avoiding liability issues. Meeting these challenges has become crucial in regards to grading food products for different markets. Food companies and suppliers need efficient, low-cost, and non-invasive quality and safety inspection technologies to enable them to satisfy different markets' needs, thereby raising their competitiveness and expanding their market share.

Quality and safety of food are usually defined by physical attributes (e.g., texture, color, marbling, tenderness), chemical attributes (e.g., fat content, moisture, protein content, pH, drip loss), and biological attributes (e.g., total bacterial count). Traditionally, assessment of quality and safety involves human visual inspection, in addition to chemical or biological determination experiments which are tedious, time-consuming, destructive, and sometimes environmentally unfriendly. These necessitate the need for accurate, fast, real-time and non chemical detection technologies, in order to optimize quality and assure safety of food.

With recent advancements in computer technology and instrumentation engineering, there have been significant advancements in techniques for assessment of food quality and safety. Machine vision and NIR spectroscopy are two of the more extensively applied methods for food quality and safety assessment. Machine vision techniques based on red-green-blue (RGB) color vision systems have been successfully applied to evaluate the external characteristics of foods [1–6]. Normal machine vision systems are not able to capture broad spectral information which is related to internal characteristics, hence computer vision has limited ability to conduct quantitative analysis of chemical components in food. Spectroscopy is a popular analytical method for quantification of the chemical components of food. The tight relationship between NIR spectra and food components makes NIR spectroscopy more attractive than the other spectroscopic techniques. However these spectral methods were proved inefficient when it comes to heterogeneous materials such as meat, owing to the fact that they are not capable of obtaining any spatial information about objects [7–10]. To solve the problem, repeated detection or ground of objects were recommended, which would raise the error or make the techniques destructive.

**Figure 1.**
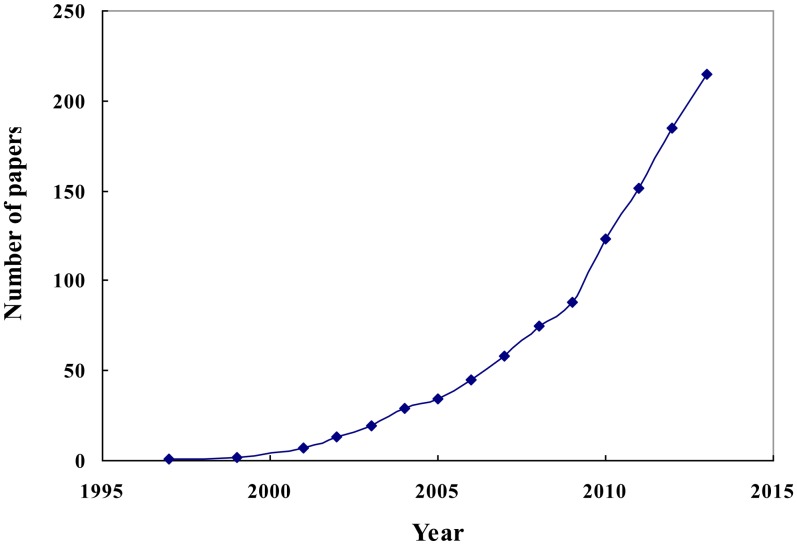
The number of publications about hyperspectral imaging applications in food.

Due to the limitations of regular machine vision and spectroscopic techniques, hyperspectral imaging was developed. Hyperspectral imaging was originally developed for remote sensing applications [11]. It can be used to obtain spectral and spatial information of an object over the ultraviolet, visible, and near-infrared spectral regions (300 nm–2,600 nm) [12]. According to Gowen *et al.* [13], hyperspectral imaging has several merits over RGB imaging, NIR spectroscopy and multispectral imaging, including the ability to collect large and detailed spectral and spatial information. Because of the inherent merits of this technique, it has been put to application in a number of fields including agriculture [14,15], pharmaceutical [16,17], and material science [18]. Applications of hyperspectral imaging in food quality and safety include detection of contaminations [19,20], identification of defects [21,22] and quantification of constituents [23]. Recently, the technique has become more and more popular in food quality control in order to meet consumer demands and the challenge of market segmentation and legal restrictions. Publications in this research area have greatly increased in number since 2008, as shown in Figure 1, which implies the strong potential of hyeprspectral imaging as a promising detection technique for food quality and safety control.

In this paper, a comprehensive review of the recent developments in hyperspectral imaging systems and applications in food and food products is provided. Compared to other recently published review articles [24–27] which focused on the applications of hyperspectral imaging in food quality inspection, this paper highlights the optical fundamentals of hyperspectral imaging and the most recent advances in the configurations and applications of hyperspectral imaging in food quality and safety control.

## 2. Hyperspectral Imaging

### 2.1. Optical Fundamentals of Hyperspectral Imaging

At the molecular level, all food samples continuously emit and absorb energy by lowering or raising their molecular energy levels. The wavelengths at which molecules absorb, reflect, and transmit electromagnetic radiation are characteristics of their structure [28]. Electromagnetic waves usually include ultraviolet radiation (UV), visible light (VIS), NIR, mid-infrared, and far-infrared (FIR). Each region is related to a specific kind of atomic or molecular transition corresponding to different frequencies. As with any biological material, food tissues are held together by several different molecular bonds and forces. Water, carbohydrates and fats are rich in O-H or C-H bonds. Organic compounds and petroleum derivatives are rich in C-H or N-H bonds. When a food sample is exposed to light, electromagnetic waves are transmitted through it, the energy of incident electromagnetic wave changes because of the stretching and bending vibrations of chemical bonds such as O-H, N-H and C-H. This makes spectroscopy able to provide characteristic and detailed fingerprints of food samples by using these observed changes in molecular energy levels.

At the macro level, the electromagnetic wave is observed as light, and the transitioning of the incident electromagnetic wave is shown as the reflection, scattering, and transmission of light. Since the absorbed part of light penetrates into the tissue of samples, the strength and wavelengths of emission and absorption depends on the physical and chemical states of the objective material. The emerging light obtained is converted to a spectrum and reshaped to images by hyperspectrometers with high signal-to-noise ratios. These obtained images, *i.e.*, hyperspectral images, could indicate the chemical constituents and physical properties of the food samples.

### 2.2. Acquisition of Hyperspectral Images

Hyperspectral imaging systems provide hyperspectral images consisting of numerous spatial image planes of the same object at different wavelengths. The resulting hyperspectral image is achieved through the superimposition of the spatial images collected by the hyperspectral sensors, thus creating a three-dimensional data cube called *hypercube* which is then further analyzed and illustrated. These images are composed of vector pixels, and represent the composition and appearance of that particular food sample. Spectra from the data cube of different samples can be compared. Similarity between the image spectra of two samples indicates similarity of chemical composition and physical features. The *hypercube* usually can be constructed in three ways: *area scanning*, *point scanning*, and *line scanning* [13]. Due to the presence of conveyor belts (for in-line inspection) in most food processing plants, *line scanning* (or *pushbroom*) is the preferred image acquisition method. The *hypercube* of *line scanning* is acquired by composing several whole lines of an image instead of a single pixel at a time, and it is stored in the format of Band Interleaved by Line (BIL) which is a scheme for storing the actual pixel values of an image in a file band by band for each line or row of the image. The spatial and spectral information stored in BIL are analyzed simultaneously.

Hyperspectral imaging systems can be operated either in reflectance or transmittance modes. To acquire images in transmittance mode, thin sample sizes are usually used to allow light to travel through the sample. Thicker samples can be used in reflectance hyperspectral imaging measurements. Thus, food materials can be inspected as a whole in reflectance mode without the need to make slices. Examples include apples [29], cucumbers [30], mushrooms [31], and chickens [32].

Light penetration depth is defined as the depth at which the incident light was reduced by 99%. It can vary according to the status, type of sample, and the detection waveband. Optical features of the light penetration depths are mainly determined by strong absorbing constituents in the sample. Research regarding the penetration depth of light in VIS and NIR range is very limited. Lammertyn *et al.* [33] proved that light penetration depth in apples was dependent on the detection wavelength by putting forward a non-linear model describing the correlation between the reflectance and thickness of apple slices. The penetration of apple is up to 4 mm in the 700–900 nm range and between 2 and 3 mm in the 900–1,900 nm range. In the research of Qin and Lu [34], the light penetration depth in tissues of apple, peach, pear, kiwifruit, tomatoes, zucchini, cucumber, and plum were calculated according to the absorption and reduced scattering spectra of the test samples at different wavelengths. The minimum light penetration depths ranged from 7.1 mm at 535 nm for the plum to 13.8 mm at 720 nm for the zucchini. The wavelengths were correlated to the absorption peaks of the major pigments in the fruits and vegetables. The maximum penetration depths ranged from 18.3 mm for the apple to 65.2 mm for the zucchini. This study highlighted that penetration depth varies a great deal depending on the type of object being studied and the applied depth. The penetration depth could have effects on the hyperspectral imaging detection. Most of the studies about penetration depth were conducted on fruits. Further research concerning penetration depth would prove beneficial by providing references for thickness determination and could be valuable for designing an appropriate and accurate sensing configuration, especially in meat products.

### 2.3. Configuration of Hyperspectral Imaging System

**Figure 2.**
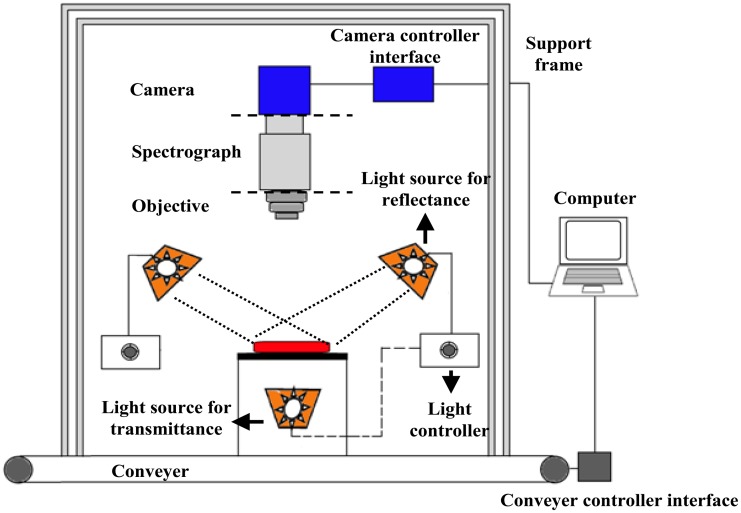
Configuration of a hyperspectral imaging system.

Typical hyperspectral imaging systems comprise hardware and software. The specific configuration may vary depending on the object to be assessed and the image acquisition technique used. Most hyperspectral imaging systems hardware platforms share common basic components (shown in Figure 2): a light source to provide illumination, usually produced by halogen-tungsten lamps; light irradiation of samples either directly or delivered by an optical fiber; a detector which obtains both spectral and spatial information simultaneously; a hyper-spectrograph to disperse the wavelengths of the reflected, transmitted, or scattered light and deliver signals to the photosensitive surface of the detector; an objective lens to adjust the range of light acquisition; an objective table fixed to a conveyer belt to hold and transport the sample and finally a computer to compose and store the three-dimensional hypercube.

**Figure 3.**
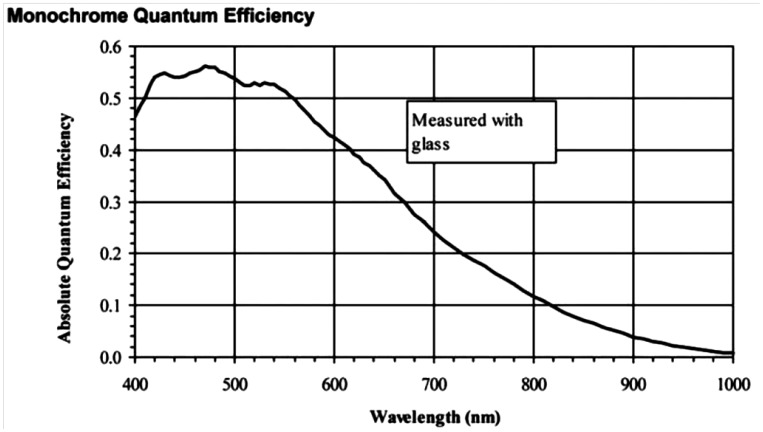
QE of typical Si based camera.

For the hyperspectral imaging system detector, there are three basic choices of cameras for this application, including silicon (Si)-based charge-coupled device (CCD) or complementary metal oxide semiconductor (CMOS) cameras, indium gallium arsenide (InGaAs)-based array detectors, and mercury cadmium telluride (HgCdTe)-based array detectors. The choice of the camera in a particular hyperspectral imaging system depends on the required wavelength, the quantum efficiency (QE) representing the sensitivity, and the cost. At present, the CCD camera (300–1,100 nm) is the most widely used VIS/NIR detector in food quality and safety analysis, with the advantage of lower cost and potential wider availability (compared to InGaAs and HgCdTe). The QE of a typical Si based sensor is shown in Figure 3. The higher QE indicates higher sensitivity. The QE of Si cameras between 420 and 560 nm is above 50%, but falls to less than 1% over 1,000 nm. This strongly indicates that to use these sensors for imaging in the NIR region, a powerful light source is required to offer very powerful output, which can be extremely expensive and run the risk of overheating the samples during imaging.

**Figure 4.**
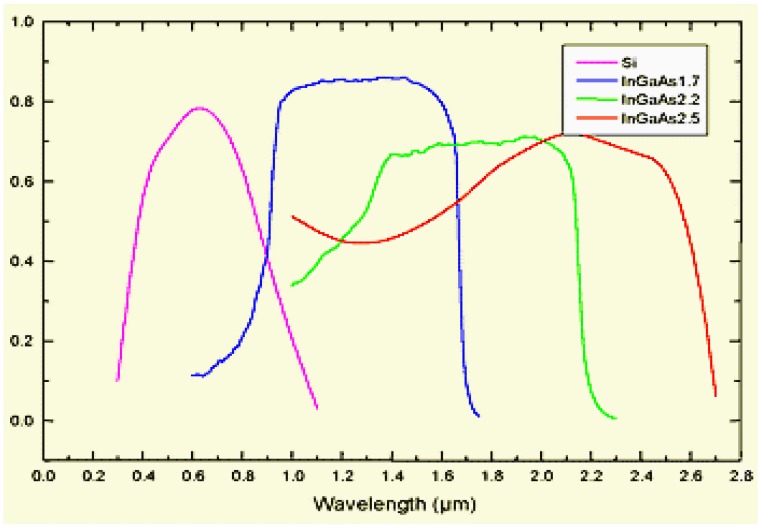
QE comparison of InGaAs detectors and Si-based cameras.

The development of advanced instrumentation enabled the application of near infrared cameras in food processing, including the InGaAs array detector (900–1,700 nm, 1,000–2,200 nm, and 1,200–2,500 nm) and the HgCdTe array detector (1,000–2,600 nm). Figure 4 shows a comparison of the QE of Si-based and InGaAs-based detectors. Three types of InGaAs detectors were studied, including InGaAs1700 (900–1,700 nm), InGaAs2200 (1,000–2,200 nm), InGaAs2500 (1,200–2,500 nm). An intersection point of the QE curves of InGaAs and Si based cameras was observed around 900 nm. The QE of InGaAs1700 falls to under 40% below 900 nm, while the QE of Si based camera falls to 40% above 900 nm. If the required wave band is around 900 nm, the choice of camera may depend on whether it tends to VIS or NIR. In contrast of three types of InGaAs detectors, a better choice would be the InGaAs1700, whose QE is higher than 50% above 950 nm and keep higher than 80% between 1,000–1,600nm, with an average QE of 60%. The InGaAs2200 and InGaAs2500 have average QE around 50% and 55% separately at the desired wavelength ranges, but not as good as InGaAs1700. All types of InGaAs cameras have better sensitivity than Si-based cameras in the NIR region especially above 900 nm. Hyperspectral imaging systems based on InGaAs camera may provide increased accuracy for assessment and analysis of food quality and safety [30,35,36], but the cost of NIR cameras is higher than for VIS/NIR cameras, which may affect the application of NIR hyperspectral imaing.

The hyperspectral imaging system and components have developed along with imaging and instrument techniques. As these technologies advance, it is expected that there will be further improvements in hyperspectral imaging systems, particularly at the higher wavelengths for more detailed analysis of food quality and safety. Very recently, a hand-held hyperspectral imaging system (400–720 nm) was proposed to assist in food processing facilities [37]. This system can be used to detect wear on HDPE surfaces as well as the presence of produce residues. Small amounts of juice released during the processing procedures of honeydew or cantaloupe melon can be detected using this handheld hyperspectral imaging system. This suggested that portable hyperspectral imaging equipment would be valuable in food quality and safety control.

### 2.4. Spatial Resolution of Hyperspectral Imaging System

The information that is acquired by a hyperspectral imaging system carries spatial information as well as spectral information. Spatial resolution is important for adjustment of the field of view and estimation of the scanning limit. From a practical point of view, a system with a proper spatial resolution should be selected according to the size and shape of the analyzed objects. Generally, the spatial resolution can be calculated by dividing the scanned spatial distance to the pixel numbers in each image. For *point scanning* system, the images are collected pixel by pixel. The spatial resolution depends on the pixel resolution of the camera. Similarly for an *area scanning* system, the images are collected area by area. The spatial resolutions in two spatial dimensions are the same. They are determined by the size of the detected area. For *line scanning* system, the dominated resolution refers to the one in the direction parallel to the slit, which is determined by several factors including zoom amount of lens, working distance, camera, imaging spectrograph, *etc.* [27]. In the study of Lara *et al.* [15], spatial resolution of 0.26 mm/pixel was used to study the shelf-life of spinach using a *line scanning* system. Kamruzzaman *et al.* [38] used a spatial resolution of 0.578 mm/pixel for visualization of minced lamb meat. Mendoza *et al.* [39] applied spatial resolution of 0.20 mm/pixel for image acquisition of apple fruit. For *line scanning* systems, different spatial resolutions were used in different studies and the most used one is at the millimetre level which was implied as the limitation of spatial resolution for *line scanning* system. It would be beneficial for food quality control if the spatial resolution of *line scanning* hyperspectral imaging could be reduced to the micron level.

## 3. Analysis of Hyperspectral Images

**Figure 5.**
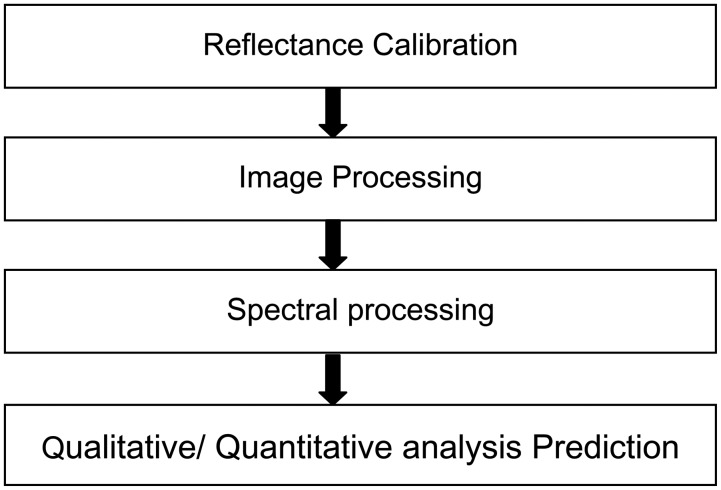
Flow diagram of hyperspectral data analysis process.

The data cube produced by hyperspectral imaging systems contains a mass of information with large dimensionality. The main purpose of hyperspectral data analysis is to reduce the dimensionality and retain the useful data for discrimination or measurement analysis of food quality and safety. Corresponding to image processing technique and chemometry, many methods could be adopted to reach the detection goal. There is a main criterion that these methods should follow as Figure 5 describes, including reflectance calibration, image processing, spectral preprocessing, and qualitative analysis or quantitative analysis.

### 3.1. Reflectance Calibration

The purpose of reflectance calibration is to correct the acquired sample images from the dark current of the camera. The dark response *D* is the background response of camera caused by dark current of the instrument. The dark response is obtained by turning off the light source, completely covering the lens with its cap, and recording the camera response. The bright response *W* representing the total reflected light intensity from the illumination is obtained from a uniform high reflectance standard-a white ceramic tile, which reflects 99% light. After the optical reflected signal *I* of sample is measured, the corrected reflectance value *R* is calculated on a pixel-by-pixel basis as follows:

(1) $$R=\frac{I-D}{W-D}$$

### 3.2. Image Processing

In the hyperspectral data cube, grayscale images with intensity scaling have different values in different pixels. These values are commonly used to display the compositional contrast of measured objects. In a hyperspectral data cube, the intensity of every pixel in the hyperspectral image represents different light reflectance or transmittance. After the signals are calibrated by Equation (1), several image preprocessing sequences should be carried out to provide greater contrast between distinct regions of the sample and the background. The typical image preprocessing techniques include edge detection techniques, filters, trend removals, band ratio, grey-level segmentation or thresholding techniques, digital morphology, texture, thinning and skeletonization algorithms [40], *etc*. Thresholding is widely used as it is necessary to segment the targeted object from the image. Region of interest (ROI), excluding redundant background in combined or original calibrated image is obtained after image preprocessing. Image preprocessing is crucial in both spectral and image processing since the selected ROI is the basis of all followed analysis.

After image preprocessing, many image analysis techniques can be used to extract useful image features for the further analysis. These include principal component analysis (PCA), minimum noise fraction, Gabor filter, wide line detector, grey level co-occurrence matrix (GLCM), variogram analysis, wavelet transform, etc. Some studies combined two or more methods to extract features more effectively [41–43].

**Figure 6.**
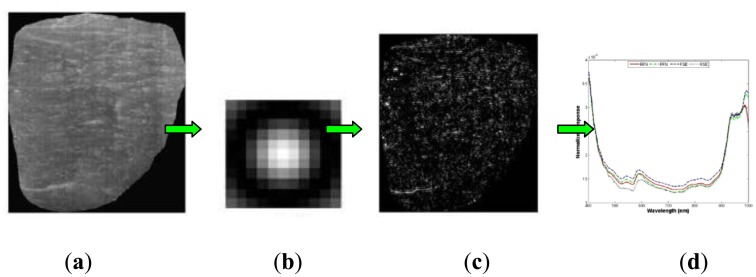
Gabor filter for extracting texture features from a ROI of a pork image. (**a**) Selected ROI of pork, (**b**) applied Gabor filter, (**c**) Gabor filtered image, (**d**) extracted texture features.

Due to the abundance of information provided by three dimensional hyperspectral data, it is necessary to extract the important features on the sample images and to compare those feature with targeted features of objects. Various feature extraction methods have been developed for feature detection and extraction in hyperspectral image processing. One example is the 2D Gabor filter. This method involves a Gaussian function modulated by a circularly symmetric sinusoidal function or oriented harmonic function, by which the spatial frequency and directional information of image texture are embodied. The Gabor filter technique was successfully applied to extract texture features from pork [41] and egg images [43] for pork quality classification and early embryo development detection, respectively. Figure 6 demonstrates an example of using Gabor filters to extract the texture feature from hyperspectral images of a pork image. ROI of pork was preliminary selected and applied to remove the useless information from original pork image. Gabor filters were performed on the hyperspectral images of the tested pork to extract the textural feature and obtain the filtered image. Finally, averaging was applied to images at all wavelengths to calculate the filtered mean spectra (shown as a plot). Useful information could be obtained by Gabor filters, which however, does not exclude redundant information. Hence, PCA, which is a technique that reduces the redundant features, was applied in the study of Liu *et al.* [41]. The principle of PCA was explained by Qin *et al.* [27]. PCA has been widely used to reduce dimension, compress data, extract feature, and even identify key wavelengths in the application of hyperspectral imaging for food quality and safety control [38,42,44–52].

**Figure 7.**
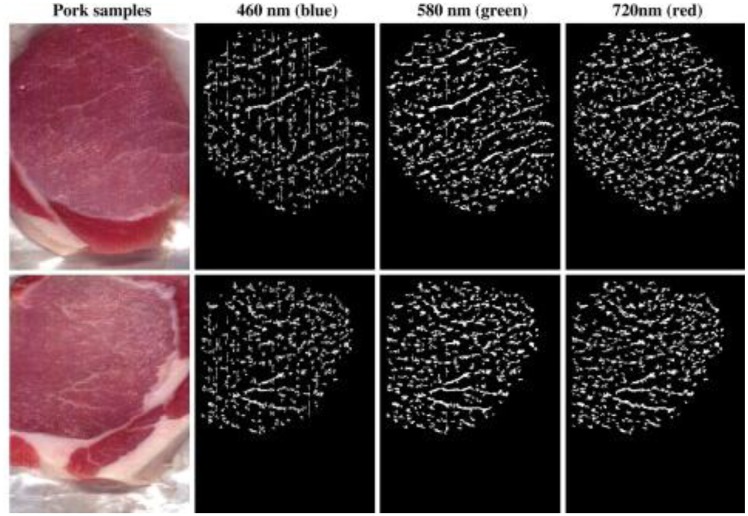
Wide line detector for extracting line feature from red, green, and blue images of pork.

Another method namely wide line detector was applied to extract the line feature from red-green-blue images of pork. The red, green, and blue images were acquired using a hyperspectral imaging system. Figure 7 shows examples of marbling extraction results, where lines with different width were efficiently extracted by wide line detector. According to Liu *et al.* [53], the wide line detector is insensitive to contrast between the object and surrounding pixels. Therefore, wide line detector is able to extract either narrow or wide lines from an image. The study is a good example of the application of pattern recognition techniques for hyperspectral analysis.

### 3.3. Spectral Processing

The basic spectral processing is spectral averaging. A mean spectrum is calculated by averaging the value of pixels included in the ROI. Individual output, *i.e.*, an individual spectrum, corresponds to individual samples. Interference signal (baseline drift, particle deviation, surface heterogeneity) could exist in spectrum. Therefore, spectral preprocessing techniques should be used to remove these nonchemical biases from the spectral information. There are a number of preprocessing techniques in spectral processing, including Savitzky-Golay filter, multiple scattering correction (MSC) for particle scattering effect elimination, first or second derivative, smoothing, and standard normal variate (SNV) [54]. Signal to noise ratio could be strengthened and more effective signals could be distinguished by computing correction factors using different preprocessing techniques. Recently, a preprocessing method named Modified Lorentzian Distribution (MLD) function, which was proposed by Peng and Lu [55], was applied to create a curve fit for beef spectral information [29], by which the effective scattering information of beef tenderness and pH value was magnified and the prediction result was improved effectively.

### 3.4. Qualitative Analysis and Quantitative Analysis

Application of hyperspectral imaging in practice may be limited due to the resulting large and computationally excessive *hypercube*. Thus, it is necessary to extract the characteristic wavelength by operating qualitative or quantitative analysis. These analyses aim to identify or explore the relationship between food features and spectral characteristics. Another important part of spectral processing is the optimization of wavelengths. For qualitative analysis, discriminate analytical tools such as manual observation, principle component analysis (PCA), linear discriminant analysis (LDA), and *k*-means clustering are usually employed to classify or evaluate samples according to a selection criterion [26,56]. Recently, Ariana and Lu [30] applied a hybrid approach, namely partial least squares discriminant analysis (PLSDA), which employs both partial least squares regression (PLSR) and LDA, to extract hyperspectral feature of defective cucumber and pickles (R > 0.80). In this study, K-nearest neighbor (KNN) was also applied to compare with PLSDA and the result indicated that PLSDA outperformed KNN. There were other reports of employment of PLSDA in image analysis of food, including pork, pickling cucumber, smoked salmon, and spinach leaves [57–60]. PLSDA was successfully practiced in those studies. However, the stability of PLSDA may be considered in further assessment [14].

Another research group [61] developed a spectral information divergence (*SID*)-based classification algorithm. *SID* is a complex value calculated from *X* and *Y*:

(2) SID(X,Y)=D(X‖Y)+D(Y‖X)

where *X* is a given hyperspectral pixel from the hyperspectral image; Y is another pixel with probability vector formed by matrix transformation from *X* [62]. After SID quantified the spectral discrepancy by making use of the relative entropy to account for the spectral information provided by each pixel, classification algorithm was applied to SID map to distinguish normal citrus from cankered citrus.

For quantitative analysis prediction, multivariate analytical tools such as PCA, PLSR, stepwise multi-linear regression (SMLR), are usually employed for chemical content prediction [27,63]. PCA and PLSR are the mostly used modeling method. Another method, support vector machines (SVM) were applied in some studies of non-invasive food quality and safety control [14,40,64]. A research group practiced radial basis function (RBF) based on least square support vector machines (LS-SVM) in hyperspectral image processing [65]. As an improvement of support vector machines (SVM), LS-SVM was showed to have better performance. This indicated that LS-SVM is an efficient quantitative analysis tool for high dimensional data.

## 4. Application of Hyperspectral Imaging in Food Analysis

As an emerging process analytical tool, hyperspectral imaging is well suited for food quality and safety control. Rapid detection and monitoring of food quality and safety are required for online implementation in food processing system. hyperspectral imaging could be used as a powerful tool for the identification of key wavelengths in the development of online automated multispectral imaging systems. Consequently, hyperspectral imaging finds widespread use in research mainly as a tool to develop multispectral inspection equipment.

Table 1 presents a summary of typical papers published in hyperspectral detection of food quality and safety since 2008. Over the past several years, intensive research has been carried with regards to the emerging potential for hyperspectral imaging application in the food industry. As shown in Table 1, reflectance mode and VIS/NIR (400–1,000 nm) region are the major application areas, while transmittance mode and NIR (900–1,700 nm) region is increasingly used to monitor external or internal features of different types of raw and processed food. Although the majority of publications on hyperspectral imaging were on fruits and vegetables, there have been growing reports of work on seafood and meat. Table 1 lists the recent advances in the application of hyperspectral imaging on quality and safety analysis of different food products.

### 4.1. Fruit

Most of the products that have been studied with hyperspectral imaging are fruits, targeting apple, citrus, pear, peach, oranges, almond nut, blueberry, citrus, grape seed, grape skin, and strawberry. The majority of these studies were carried out in reflectance mode and in the VIS-NIR range (about 400–1,100 nm). Chilling defects, diseases and quality attributes of fruits including soluble solids content, mealiness, *etc.*, were clarified. Besides, a few studies have been carried out on strawberries and grape seeds in the NIR range (about 900–1,700 nm) [49,100,101].

One quality attribute of fruit that has been assessed using hyperspectral imaging is soluble solids content. Peng and Lu [29] designed a reflectance system to detect apple firmness and soluble solids content using stable object stage. Optic fiber and focusing lenses were used to illuminate samples as a spot light source and 2D hyperspectral images were collected. The light source used in this study was delivered by a circular beam of 1.5 mm, which scanned the fruit 1.6 mm off the incident center. Ten MLD functions were proposed to fit the spectral scattering profiles and the best one was chosen as the ideal method for predicting fruit firmness and SSC using MLR. The best prediction results with two attributes of apple were got with correlation coefficient of 0.85. In the study, over 20 wavelengths were used for prediction. This may influence the data processing speed. Later, Mendoza *et al.* [39] employed integrated spectral scattering and image characteristics to predict the firmness and soluble solids content of apples. The results indicated an increase of 6% of standard errors of prediction (SEP) for firmness and 3% of SEP for soluble solids content. Large latent variables were adopted in the prediction model, which indicated the necessary of a more robust prediction model for firmness and soluble solids content of apples. Leiva-Valenzuela *et al.* [74] used VIS/NIR hyperspectral imaging (500–1,000 nm) to determine the soluble solids content in blueberries, reaching prediction accuracies of 0.87 and 0.79 for firmness and soluble solids content, respectively.

Another quality attribute of fruit evaluated using hyperspectral imaging is mealiness. Huang and Lu [66] examined the relationship between reflectance hyperspectral line images and apple mealiness. The spectral scattering profiles at individual wavelengths of apples undergoing different time, images were obtained and correlated to different mealiness levels. The mealiness of the apple was determined by the hardness and juiciness. Its correlation with hyperspectral scattering profiles was predicted using PLS. Classification models with two-class or more class were built using PLSDA. The best classification accuracy was obtained in the classification of ‘non mealy’ and ‘mealy’ apples, with an accuracy of 75%. This study demonstrated that hyperspectral scattering technique was potentially useful for nondestructive detection of apple mealiness and suggested that further research should focus on improving the classification accuracy especially for discrimination of less severe mealy apples. The same spatially resolved diffuse reflectance hyperspectral imaging system was used to study optical properties of fruits and vegetables including apple, pear, cucumber, tomato [34]. The study reinforced hyperspectral imaging technique's potential as a convenient attribute classification means for many fruits and vegetables. In study of Huang *et al.* [68], the mealiness of apple was determined using VIS/ NIR hyperspectral imaging. A classification accuracy of 82.5% was obtained using LLE algorithm-assisted SVM models.

Some work also has been done for defect detection of fruits. Qin *et al.* [61] examined the relationship between hyperspectral area images and citrus canker, using an Electron-Multiplying Charge-Coupled-Device (EMCCD) imaging device which is a type of CCD with high photosensitivity. Later, the hyperspectral area images were processed and classified to differentiate citrus canker lesion from normal and other peel diseased conditions including greasy spot, insect damage, melanose, scab, and wind scar. The analysis method was SID based classification. The overall classification accuracy was 96%. It was noted that canker lesions at different developmental stages affected the SID-based classification results. Indeed, canker lesions influenced the reflectance characteristics of a given object. Further work should be targeted at the changing patterns of the citrus canker reflectance properties and incorporate canker spectra at different growth stages. Meanwhile, since this research used full spectral information which was not good for online citrus canker detection, more work could be done at exploring better method to optimize waveband combination and raise image processing speed. Further research should concentrate on improving the processing speed of the large amount of hyperspectral information.

### 4.2. Vegetables

The main application of hyperspectral imaging on vegetables includes onions, mushrooms, pickling cucumbers and whole pickles, spinach leaves, and cherry tomato. The major adopted mode is still reflectance mode, while few studies were conducted in transmittance model and fluorescence modes.

Ariana and Lu [93] developed a VIS-NIR hyperpsectral imaging system combining reflectance mode and transmission mode together, while using a moving transport platform. This system was applied to detect inner defected pickle pieces and classify pickling cucumbers and pickles, with spectral range of 500–1,000 nm. The system was capable of identifying inner defects of cucumber and pickles which were invisible to the naked eye.

Six papers have been published on mushroom quality detection using hyperspectral imaging. Taghizadeh *et al.* [81] investigated the shelf life (using parameters including weight loss, color, maturity index) of mushrooms under different packaging polymer films (polyvinyl chloride (PVC), polyethylene terephthalate (PET) with different levels of perforations). This research demonstrated that hyperspectral imaging has potential as an analytical tool for evaluation of shelf-life of fresh mushrooms. It also indicated hyperspectral imaging can be used to evaluate the effect of different packaging solutions especially packaging materials. It indicated that PET packaging film perforated with diameter 1mm was generally superior and viable alternative to PVC film in terms of maintaining overall mushroom quality.

The bruise damage [83] and freeze damage [31] of mushroom were identified using PCA. Mushroom slice quality was measured in terms of moisture content, colour and texture [31,83,84] using MLR and principal components regression (PCR). It is resulted that hyperspectral imaging is potential for damage detection and quality measurement of mushroom.

Gaston *et al.* [82] concerned with the prediction of polyphenol oxidase (PPO) activity on mushroom. PCA was used as a data analysis method. The result of this study revealed the possibility of developing a sensor that could rapidly identify mushrooms with a higher likelihood to develop enzymatic browning. Indeed, this study highlights the utility of hyperspectral imaging in terms of safety and quality management in the food industry.

### 4.3. Meat

Most meat researches related to hyperspectral detection were performed on pork, beef, and chicken fillet. Lamb [46] and ham [47,79] were also investigated. Liu *et al.* [41] applied a Gabor filter which is used in pattern recognition to preprocess hyperspectral images of pork. PCA was used to compress spectral features over the entire wavelengths (400–1,000 nm) into principal components (PCs). ‘Hybrid’ PCs were created by combining PCs from hyperspectral images with PC(s) from Gabor-filtered images. Both K-means clustering and LDA were applied to classify pork samples. The overall unbiased statistical classification accuracy reached 84 ± 1%. The comparison results of hyperspectral images and Gabor-filtered images based analysis proved that the texture features extracted by Gabor filter offered useful information for the differentiation of different levels of pork quality.

Liu *et al.* [53] proposed an automatic and objective evaluation method for pork marbling score assessment in the view of pattern recognition. The wide line detector which is adopted in pattern recognition was applied for marbling extraction. Standard charts of marbling scores were used. The seven levels of marbling score were classified with an accuracy of 99%. The data used by Qiao *et al.* [23] were employed in this study to investigate marbling score estimation. The assessment result was much more accurate than Qiao's result in which the texture indices were extracted from the hyperspectral images by co-occurrence matrix. This study alleviated the contrast problem brought about by PSE and PFN samples when subjective marbling assessment was made on these pale and reflective pork samples. Huang *et al.* [99] studied the wide line detector and Gabor filter for NIR spectral image analysis of pork marbling. It turned out that the Gabor filter outperformed the wide line detector for processing of NIR images. Huang *et al.* [98] applied a Gabor filter and GLCM to determine the intramuscular fat content in fresh loin cut. The best results of correlation coefficients of calibration and cross validation (0.89 for both) were obtained for non-destructive prediction of IMF content of intact pork using the Gabor filter. The results of these studies implied that hyperspectral imaging has great potential to predict the fat attributes of pork. Proper image processing technique could improve the accuracy of estimation.

Peng *et al.* [65,70] studied the bacterial spoilage process in beef and pork, respectively using total NIR/VIS reflectance hyperspectral imaging system (400–1,000 nm). The best prediction result (correlation coefficient = 0.95, standard error of prediction (SEP) = 0.30) was obtained using combination of scattering parameters. These two studies demonstrated the great potential of hyperspectral imaging in bacterial activity which causes quality change of food. Multi-linear regression (MLR) using SMLR selected waveband combination was preferable to separate fresh beef and unfresh beef, while LS-SVM was the preferred method used to detect pork storage time. It was shown that hyperspectral imaging can provide quantitative information for bacterial concentration in beef and pork samples. The lowest bacterial concentration in beef of the study above was 1 × 10^4^/g, which showed that NIR/VIS spectral signal were ideal for detection of bacteria concentration (≥1 × 10^4^). The results of these two researches also noted that hyperspectral imaging could be used to predict the shelf life or the storage time of beef or pork.

Barbin *et al.* [85] studied the grading and classification of pork using near-infrared hyperspectral imaging (900–1,700 nm). Different to the study of Qiao *et al.* [23], three quality grades (PSE (pale/pinkish-gray, soft and exudative), RFN (reddish-pink, firm and non-exudative) and DFD (dark purplish red, firm and dry)) were studied as grades of pork. Obvious reflectance differences of 2nd derivative spectra among the pork of three quality grades were observed at wavelengths 960, 1,074, 1,124, 1,147, 1,207 and 1,341 nm. Principal component analysis was carried out and the accuracy was 96%. The results of this study indicated that pork classes could be precisely discriminated using NIR hyperspectral imaging. More work could be conducted to classify fours types (PSE, RFN, RSE (reddish-pink, soft and exudative), PFN (pale/pinkish-gray, firm and non-exudative)) of pork totally.

Different types of lamb muscles from different parts of Charollais breed were imaged and analyzed using PCA in study of Kamruzzaman *et al.* [46]. The results demonstrated the potential of hyperspectral imaging in quality inspection of lamb.

Another developing direction of application of hyperspectral imaging is the visualization of prediction/ distribution map of quality attributes in the tested food. Since the hyperspectral images combined spectral of each pixel and image at each wavelength together, it is convenient to generate the distribution map using the built prediction models by extracting the feature of each pixel and inputting them to the corresponding prediction model. Successful examples were demonstrated for visualization of L* values (the lightness of the colour), pH values, and drip loss in pork, Enterobacteriaceae on chicken fillets, L* values in lamb, adulteration at different levels in pork [38,76,78,86].

### 4.4. Seafood

Few studies on seafood have been reported in the last few years. The tested samples included fresh and smoked salmon, cod, prawn, and shell-free cooked clams. Considering the difficulties caused by shells, seafood holds promise as an attractive area for hyperspectral imaging research. The mostly studied object is salmon, whose fillet remains smooth and shell-free. Huang *et al.* [89] applied hyperspectral imaging on salmon's storage time prediction. PCA based *K*-means clustering and MLR were applied to relate hyperspectral data to the storage time and texture change of salmon, respectively. The result indicated that it is possible to predict the texture and storage time using hyperspectral imaging. Wu *et al.* [90] used hyperspectral imaging to measure the color distribution in salmon fillet. Successive projections algorithm was employed to select effective wavelengths. Correlation coefficients of 0.876, 0.744, and 0.803 were obtained for L*, a*, and b* (three coordinates of the Lab colour space representing the lightness of the colour, its position between red/magenta and green and its position between yellow and blue), respectively. Ivorra *et al.* [59] studied the potential of NIR hyperspectral imaging for detection of expired vacuum-packed smoked salmon. The classification success rate of 82.7% demonstrated the potential of hyperspectral imaging as a commercial tool for identification of expired salmon.

VIS/NIR hyperspectral images were investigated by Wu *et al.* [87] to detect the gelatin adulteration in prawn. The combination of uninformation variable elimination and successive projections algorithm (SPA) was applied for the first time to select the optimal wavelengths in the hyperspectral image analysis. A coefficient of determination of 0.965 was obtained and gelatin in all portions of the prawn was visualized. Coelho et al. [106] used hyeprspectral imaging to detect the parasite in the shell-free cooked clam *Mulinia edulis*. Transmittance mode was used in this study. The range of wavelengths between 600–950 nm was identified to be sensitive, where changes were observed in the normalized optical responses of clam's mantle cavity in condition of parasite. Reduction in the normalized transmittance of clam's mantle indicated a hidden parasite inside the clam. Transmittance features over 720 nm achieved a 100% detection accuracy. Normalized transmittance was suggested to be a proper feature for development of non-destructive parasite-detector. Further work was suggested to develop a methodology for wavelength selection in predefined conditions.

### 4.5. Grains

Hyperspectral imaging has been applied for classification of grains including maize, wheat, barley, oat and groat, soybean, and rice seed. A NIR hyperspectral imaging system (900–1,700 nm) was developed in a mathematical modeling framework to identify pregerminated barley at an early stage in order to segregate the barley kernels into low or high quality [45]. This system employed a supervised classification framework based on a set of features which are insensitive to the kernel orientation. A low classification error of 3% proved feasibility of the developed system for describing the degree of pregermination of single barley kernels. Williams *et al.* [36] described the application of InGaAs and HgCdTe detector-based NIR hyperspectral imaging (960–1662 nm) techniques in distinguishing between hard, intermediate and soft maize kernels. PCA and multivariate data analysis were applied to detect glassy (hard) and floury (soft) endosperm inside the maize kernels. It resulted that the InGaAs detector-based NIR hyperspectral imaging system obtained a better coefficient of determination to distinguish between glassy and floury endosperm (84.9%) comparing to the HgCdTe detector-based NIR hyperspectral imaging system (76.3%). Serranti *et al.* developed an NIR hyperspectral imaging system (1,006–1,650 nm) for classification of oat and groat kernels [51]. The obtained hyperspectral images were analyzed using PCA and PLS-DA to build the classification models to discriminate oat and groat kernels. Three key wavelengths, *i.e.*, 1,132, 1,195 and 1,608 nm, were identified using a bootstrap-VIP procedure. The very high classification result (almost 100%) strongly indicated the big potential of hyperpsectral imaging in industrial application. Another NIR hyperspectral image system (960–1,700 nm) was developed for differentiation of wheat classes grown in western Canada. Different classification models were used in the system. Classification accuracies of 94%–100%, 86%–100%, and 80%–100% were obtained from LDA, quadratic discriminant analysis (QDA) and ANN models, respectively. It was concluded that NIR hyperspectral imaging along with statistical and ANN classifiers has the potential to effectively classify Canadian wheat.

Some research work also has been conducted for detection of damage and contaminants of grains. Williams *et al.* [96] developed the NIR hyperspectral imaging system (1,000–2,498 nm) to track changes in fungal contamination of whole maize kernels. PLS regression models were established to assess the changes over time. The results indicated the possibility of the early detection of fungal contamination and activity. NIR hyperspectral imaging technology has also been applied to detect damaged wheat kernels. A NIR hyperspectral imaging system in the range of wavelengths 1,000–1,600 nm was developed for detection of insect-damaged wheat kernels [103]. LDA and QDA were employed to classify healthy and insect-damaged wheat kernels and the classification accuracy was 85%–100%. Later, another NIR hyperspectral imaging system (700–1,100 nm) was established to discriminate healthy and midge-damaged wheat kernels by the same research team [92]. Statistical features and histogram features were extracted from hyperspectral images at significant wavelengths. Three statistical classifiers were used for classification. The high average accuracy (95.3%–99.3%) strongly indicated the potential of NIR hyperspectral imaging for detection of damaged wheat kernels.

### 4.6. Biofilm Detection

Recently, Jun *et al.* [107] reported the utilization of macro-scale fluorescence hyperspectral imaging to evaluate the potential detection of pathogenic bacterial biofilm formations on five types of food-contact surface materials: stainless steel, high density polyethylene (HDPE), plastic laminate (Formica), and two variations of polished granite. These materials are commonly used to process and handle food, and sometimes cause biofilm pollution on food surface. Spots of biofilm (*E. coli* O157:H7 and *Salmonella* biofilm) growth were produced on sample surfaces and stored and scanned by fluorescence hyperspectral imaging system using ultraviolet-A excitation (421–700 nm, including a C-mount object lens, F1.9 35 mm). PCA was used to reduce the dimensionality of hyperspectral images and an image processing method was developed based on single-band and two-band ratio techniques to select the wavebands appropriate for differentiating biofilm spots form different backgrounds. The suitable spectral fluorescence band for detecting microbial biofilm on stainless steel surfaces was 559 nm, with overall detection rate of 95%. For HDPE and granite, ratios between different two bands provided the most efficient results. For Formica, the results were not accurate enough to detect biofilms effectively. The result of this study showed the hyperspectral imaging could also be used to develop portable hand-held devices for sanitation inspection of food packaging, which has been a big issue for food processing. It was also noted that low cell population density may influence the accuracy of biofilm inspection of food processing surfaces. More studies could be conducted on the hyperspectral imaging biofilm detection, especially in low cell population density.

## 5. Discussion and Conclusions

Hyperspectral imaging is developing as a platform technology for food quality and safety analysis in food processing and packaging. Hyperspectral imaging could obtain the internal spectral information of samples while detecting spatial signals, which are related to the physical and chemical features of a large amount of food samples and food-contact surface materials. These signals are stored in large data cube which may slow down the data processing speed. Thus, increasing the efficiency of the identification of key wavelengths should be the center focus of upcoming studies. Improvements in the data analysis would elevate the processing speed of hyperspectral imaging data, making hyperspectral imaging more suitable for online detection, and providing the basis of multiple-spectral system production. Also, the enhancement of the sensitivity and pixel resolution of camera would help to improve the prediction accuracy of hyperspectral imaging. The achievements of the research in hyperspectral imaging strongly indicate that hyperspectral imaging, especially NIR hyperspectral imaging, has a big potential in detecting quality and safety of meat and seafood products, as well as biofilm for food packaging. More applications of hyperspectral imaging technology in food quality and safety inspection during food processing and packaging will be investigated. Future work in hyperspectral imaging could focus on issues such as higher sensitivity cameras, higher resolution systems, improvements in data processing methods, increasing detection accuracy, and expanding the range of applicable food products.

## Figures and Tables

**Table 1.** Summary of measurement mode, product type, analysis type, wavelength region, and modeling algorithm in representative papers published on hyperspectral imaging of food since 2008.

**Mode**	**Camera**	**Product**	**Spectral Coverage (nm)**	**Analysis Type**	**Image Processing**	**Modeling**	**Reference**
**Reflectance**	CCD	Almond nut	700–1,000, 950–1,390	Qualitative	Not-mentioned	Band ratio(BR), Support vector machines (SVM)	[64]
	CCD	Apple	600–1,000	Quantitative	Thresholding (TH)	Partial least squares regression(PLSR), Partial least squares discriminant analysis (PLSDA)	[66]
	CCD	Apple	450–1,000	Quantitative	Not-mentioned	Stepwise multi-linear regression(SMLR)	[29]
	CCD	Apple	400–1,000	Qualitative	TH	Artificial neural networks (ANN)	[67]
	Not-mentioned	Apple	600–1,000	Qualitative	Locally linear embedding (LLE)	SVM, PLSDA	[68]
	EMCCD	Apple	400–1,000	Quantitative	First derivative, and multi-resolution wavelet transform	PLSR	[69]
	EMCCD	Apple	500–1,000	Quantitative	First order statistics, Fourier fractal texture, grey level co-occurrence matrix (GLCM), run length matrix (RLM), directional fractal dimension analysis, and multi-resolution wavelet transform	PLSR	[39]
	Not-mentioned	Apple	400–1,000, 1,000–2,500	Qualitative	PCA, minimum noise fraction (MNF)	Soft independent modeling class analogy (SIMCA), linear discriminant analysis (LDA), SVM	[42]
	CCD	Apple, peach, kiwifruit, plum	500–1,000	Quantitative	TH	Manual analysis	[34]
	CCD	Beef	400–1,000	Quantitative	Co-occurrence matrix analysis, PCA	Canonical discriminant	[44]
	CCD	Beef	400–1,100	Quantitative	MLD	Multi-linear regression (MLR)	[70]
	CCD	Beef	496–1,036	Quantitative	MLD	SMLR	[71]
	CCD	Beef	910–1,700	Quantitative	PCA	PLSR	[72]
	CCD	Beef	897–1,752	Quantitative	TH	PLSR	[73]
	EMCCD	Blueberry	500–1,000	Quantitative	TH	PLSR	[74]
	CCD	Chicken	389–744	Qualitative	TH	BR	[32]
	CCD	Chicken breast fillets	910–1,700	Quantitative	TH	PLSR	[75]
	CCD	Chicken fillets	930–1,450	Quantitative	TH	PLSR	[76]
	CCD	Citrus	400–1,100	Qualitative	Geometric factor correction(GFC)	Digital elevation model (DEM)	[77]
	EMCCD	Citrus	450–930	Qualitative	TH	Spectral information divergence (SID) mapping	[61]
	CCD	Grape seed	914–1,715	Quantitative	PCA	PLSR	[49]
	CCD	Lamb	910–1,700	Qualitative	PCA	PCA	[46]
	CCD	Lamb	900–1,700	Quantitative	TH	PLSR	[78]
	CCD	Minced lamb	890–1,750	Quantitative	PCA	PLSR, MLR	[38]
	CCD	Ham	910–1,710	Qualitative	PCA	PCA	[47]
	CCD	Dry-cured ham	760–1,040	Quantitative	Not-mentioned	PLSR	[79]
	CCD	Mandarin	320–1,100	Qualitative	GFC	LDA, Classification and regression trees (CART)	[80]
	CCD	Mushroom	400–1,000	Quantitative	Not-mentioned	PLSR	[81]
	CCD	Mushroom	400–1,000	Quantitative	TH	PCA	[82]
	Not-mentioned	Mushroom	400–1,000	Qualitative	TH	PCA	[83]
	Not-mentioned	Mushroom	450–850	Quantitative	Not-mentioned	MLR, Principal components regression (PCR)	[84]
	Not-mentioned	Mushroom	450–950	Qualitative	Interactive selection	PCA	[31]
	CCD	Oranges	400–1,100	Qualitative	PCA	PCA, BR, TH	[48]
	CCD	Pork	400–1,000	Quantitative	Gabor-filter, TH	PCA, K-means clustering, LDA	[41]
	CCD	Pork	400-1,100	Quantitative	Not-mentioned	Least square support vector machines (LS-SVM)	[65]
	CCD	Pork	460, 580, 720	Quantitative	Wide line detector	MLR	[53]
	CCD	Pork	900–1,700	Qualitative	PCA	PLS	[50]
	CCD	Pork	900–1,700	Qualitative	TH	PLSDA	[57]
	Not-mentioned	Pork	900–1,700	Qualitative	TH	PCA	[85]
	CCD	Pork	900–1,700	Quantitative	TH	PLSR	[86]
	CCD	Pickling cucumbers and whole pickles	400–740	Qualitative	TH	PLSDA, K-nearest neighbor(KNN)	[30]
	CCD	Pickling cucumbers	450–740	Qualitative	TH	PLSDA	[58]
	CCD	Prawn	897–1,753	Quantitative	TH	Uninformation variable elimination, Ssuccessive projections algorithm,	[87]
	Not-mentioned	Rice seed cultivar	874–1,734	Qualitative	Not-mentioned	PLSDA, SIMCA, KNN, SVM, random forest (RF)	[88]
	CCD	Salmon	400–1,100	Qualitative & Quantitative	TH	PCA, K-means clustering, MLR	[89]
	CCD	Salmon	964–1,631	Quantitative	Predictive effective wavelengths (PEW)	Multiple linear regression (MLR)	[90]
	CCD	Smoked salmon	400–1,000	Qualitative	Quartiles segmentation, TH	PLSDA	[59]
	EMCCD	Spinach leaves	400–1,000	Qualitative	TH	Spectral angle mapper (SAM), PLSDA, Leafy Vegetable Evolution	[60]
	CCD	Spinach	400–1,000	Qualitative	Radiometric correction	PCA, analysis of Variance	[15]
	Not-mentioned	Wheat ears	400–1,000	Qualitative	TH	SAM	[91]
	FFT-CCD	Wheat	700–1,100	Qualitative	PCA	LDA, Quadratic discriminant analysis (QDA), Mahalanobis discriminant classifier	[92]
	CCD	Whole pickles	400–675	Qualitative	TH	PCA	[93]
	CCD	Whole grape skin	400–1,000	Quantitative	Not-mentioned	PCA, Adaboost	[94]
	InGaAs	Beef	900–1,700	Quantitative	TH, BR	PLSR	[95]
	InGaAs	Barley	900–1,700	Quantitative	PCA, MNF	Maximum likelihood multinomial regression classifier	[45]
	InGaAs, HgCdTe	Maize	960–1,662 1,000–2,498	Qualitative	TH	PLS-DA	[36]
	HgCdTe	Maize	1,000–2,498	Qualitative	PCA	PLSR	[96]
	InGaAs	Onion	1,000–1,600	Qualitative	TH	Manual analysis	[97]
	InGaAs	Oat and groat	1,006–1,650	Qualitative	PCA	PLS-DA	[51]
	InGaAs	Pork	900–1,700	Qualitative	Gabor filter, GLCM, TH	PLSR	[98]
	InGaAs	Pork	900–1,700	Quanlitative	Gaborfilter, GLCM, TH	PLSR	[99]
	InGaAs	Strawberry	1,000–1,600	Qualitative	TH	LDA	[100]
	InGaAs	Strawberry	1,000–1,600	Qualitative	Multi-band image segmentation	Multi-band multivariate classifiers, uni-band univariate classifiers, multiband decision-fusion classification	[101]
	InGaAs	Wheat	960–1,700	Qualitative	Image cropping, feature extraction	LDA, QDA, ANN	[102]
	InGaAs	Wheat	1,000–1,600	Qualitative	PCA	LDA, QDA	[103]
Transmittance	CCD	Cod	448–752	Qualitative	TH	Gaussian maximum likelihood (GML) classifier	[104]
	CCD	Egg	550–899	Qualitative	Not-mentioned	PCA	[105]
	InGaAs	Egg	900–1,700	Qualitative	TH, Gabor filter	K-means clustering	[43]
	CCD	Pickling cucumbers and whole pickles	740–1,000	Qualitative	TH	PLSDA, KNN	[30]
	CCD	Pickling cucumbers	740–1,000	Qualitative	TH	PLSDA	[58]
	CMOS	Shell-free cooked clam	600–950	Qualitative	TH	Supervised parasite detector	[106]
	CCD	Vegetable soybean	400–1,000	Qualitative	Not-mentioned	Support vector data description (SVDD) classifier	[14]
	CCD	Whole pickles	675–1,000	Qualitative	TH	PCA	[93]
Fluorescence	EMCCD	Microbial biofilm formation	421–700	Qualitative	TH	PCA	[107]
	EMCCD	Cherry tomato	400–700	Qualitative	PCA	PCA	[52]
	CCD	Maize	400–700	Qualitative	TH	Discriminant analysis	[19]
